# Development of the Penn Healthy Diet screener with reference to adult dietary intake data from the National Health and Nutrition Examination Survey

**DOI:** 10.1186/s12937-022-00821-w

**Published:** 2022-11-17

**Authors:** Charlene W. Compher, Ryan Quinn, Frances Burke, Doris Piccinin, Linda Sartor, James D. Lewis, Gary D. Wu

**Affiliations:** 1 University of Pennsylvania School of Nursing, Philadelphia, PA USA; 2 Department of Cardiology, Perelman University of Pennsylvania School of Medicine, Philadelphia, PA USA; 3 Abramson Cancer Center, Perelman University of Pennsylvania School of Medicine, Philadelphia, PA USA; 4 Bariatric Surgery Program, Perelman University of Pennsylvania School of Medicine, Philadelphia, PA USA; 5 Department of Gastroenterology, Perelman University of Pennsylvania School of Medicine, Philadelphia, PA USA

**Keywords:** Healthy diet, Screener, NHANES, HEI-2015, Food pattern, Adult

## Abstract

**Background:**

There is a need for a feasible, user-friendly tool that can be employed to assess the overall quality of the diet in U.S. clinical settings. Our objectives were to develop the Penn Healthy Diet (PHD) screener, evaluate screener item correlations with Healthy Eating Index (HEI)-2015 components, and develop a simple scoring algorithm.

**Methods:**

National Health and Nutrition Examination Survey (NHANES) 2017–18 dietary recall data in adults were used to define food examples in screener food groups based on components of the HEI-2015, Diet Approach to Stop Hypertension, and Alternative Mediterranean diet approaches. Instrument Content Validity Index (I-CVI) was used to evaluate the clarity and relevance of the screener. Patient acceptability was evaluated by completion time and response rates. NHANES 2017–18 food recall data were used to simulate responses to the screener items, which were evaluated for association with HEI-2015 components. A scoring algorithm was developed based on screener items moderately or strongly associated with HEI-2015 components. Reproducibility was tested using NHANES 2015–16 data.

**Results:**

The screener had strong clarity (I-CVI = 0.971) and relevance for nutrition counseling (I-CVI = 0.971). Median (IQR) completion time was 4 (3–5) minutes on paper and 4 (4–8) minutes online, and 73% of patients invited online completed the survey. Based on simulated NHANES participant screener responses, 15 of the 29 screener items were moderately or strongly associated with HEI-2015 components, forming the basis of the scoring algorithm with a range of 0–63 points, where higher score indicates a healthier diet. The median (IQR) screener and HEI-2015 scores were 14.96 (11.99–18.36) and 48.96 (39.51–59.48), respectively. The simulated PHD score was highly correlated with the HEI-2015 score (Spearman rho 0.75) in NHANES 2017–18 and confirmed in NHANES 2015–16 data (Spearman rho 0.75).

**Conclusions:**

The Penn Healthy Diet screener may be a useful tool for assessing diet quality due to its acceptable content validity, ease of administration, and ability to distinguish between servings of key food groups associated with a healthy versus unhealthy diet according to the HEI-2015. Additional research is needed to further establish the instrument’s validity, and to refine a scoring algorithm.

**Supplementary Information:**

The online version contains supplementary material available at 10.1186/s12937-022-00821-w.

## Introduction

Dietary patterns have been linked to many of the most common causes of morbidity and mortality in the modern world, such as metabolic syndrome, diabetes mellitus, cardiovascular disease, and cancer [1]. Metabolic syndrome is characterized by abdominal adiposity, dyslipidemia, hypertension, and insulin resistance, and is associated with cardiovascular disease, diabetes mellitus and cancer [2, 3]. The prevalence of metabolic syndrome in the United States (US) rose more than 35% between 1988 and 1994 and 2007–2012 to a prevalence rate of 34% [4]. Cardiovascular disease remains the leading cause of death in US adults, with a healthcare cost of $363 billion in 2016–2017 [5]. More than 34 million US adults have type 2 diabetes, and 88 million more have prediabetes [6]. Cancer deaths remain considerably higher than the 2030 Healthy People goal of 122.7 deaths per 100,000 population (currently 172.9 in males and 126.2 deaths per 100,000 in females respectively) [7].

Because of the association of these health conditions with dietary intake, efforts at primary and secondary disease prevention and treatment often include dietary counseling. Indeed, dietary approaches such as the Healthy Eating Index-2015 (HEI-2015) [8–11], Mediterranean [11–16], and Dietary Approach to Stop Hypertension (DASH) [11, 13, 17–22] have been associated with beneficial health outcomes. Identification of dietary patterns that correlate with health and disease may be particularly useful for diet counseling or to identify food-related characteristics of populations for research [20, 23].

Dietary intake information is essential for personalized diet counseling and for research. However, dietary intake information can be challenging to obtain due to professional time constraints and participant burden. Brief dietary intake tools such as screeners may be particularly useful to obtain a snapshot description of the overall quality of an individual’s diet [23] as a strategy to focus diet teaching or begin the conversation about dietary goals. Even though the detail provided by a screener is limited and measurement error is greater than with a series of 24-hour dietary recalls, recalls are not feasible for dietitians to obtain and analyze in real time during a counseling session. Furthermore, dietary advice is provided based on foods while nutrient-level data from computer-based analysis are less useful.

Currently available dietary screeners that were designed based on small samples or from specific geographic areas may have limited generalizability to today’s diverse US population. The Rapid Eating Assessment for Participants-shortened version (REAP-S) was designed in 2004 to assist primary care providers to advise patients on diet [24]. With questions focused on unhealthy eating behaviors, the REAP-S screener has not been aligned with nationally representative data or the HEI-2015 dietary guidelines. The Dietary Screening Tool (DST) was created in 2007 based on commonly reported food items from rural older adults in Pennsylvania who were enrolled in a Medicare health maintenance organization with a scoring algorithm loosely based on the HEI-2005 [25]. Foods eaten by racial or ethnic groups in other parts of the country or new foods entering the food supply in the past 15 years would be challenging to classify using the DST. The Mediterranean Diet Adherence Screener (MEDAS) was designed based on food patterns typical in Spain that were associated with lower cardiac risk [26]. However, the food items that are emphasized (olive oil, nuts, wine, fish) are not taken frequently by Americans, and optimal intake by MEDAS may not meet US Dietary Reference Intake (DRI) levels. The Dietary Screener Questionnaire (DSQ) based on NHANES 2009–10 dietary recall data contains 26 items [27], and asks about monthly intake with variable numbers of servings depending on the item. The complexity of variation in the frequency of servings on the DSQ and the long window of recall provide cognitive burden for participants that would be challenging in clinical contexts. A more current screener based on typical foods in the US diet that aligns with the HEI-2015 would be useful.

The development of an ideal dietary screener for use in clinical settings requires consideration of many factors [28]. The ability to measure overall diet quality, validity against another diet assessment method such as 24-hour recall, and validity within diverse group are important [28]. An ideal screener for diet counseling should identify optimal foods to obtain DRI levels, as indexed in the HEI-2015, as well as foods to discuss for management of cardiometabolic diseases. For use in a clinical setting, screeners should be brief and user-friendly, accommodate automated scoring that can be associated with clinical decision support, sensitive to change in diet over time, and useful for chronic disease management [28]. Given the need for such a dietary screener, we developed the Penn Healthy Diet (PHD) screener. Our objectives were to:

- develop and provide initial content validation for a dietary intake screening tool that would be useful to guide nutrition counseling,
- compare simulated screener responses from adult National Health and Nutrition Examination Survey (NHANES) 24-hour recall data to HEI-2015 components computed from the recalls, and
- create a simple scoring algorithm.

We hypothesized (a) that the screener would be feasible for independent administration and useful for diet counseling in clinical settings, (b) that simulated responses to screener items would correlate with HEI-2015 components, and (3) that the screener scoring algorithm would be correlated to items in the HEI.

## Materials and methods

The project was considered quality improvement for patient care and determined not to require formal IRB review. The National Health and Nutrition Examination Survey is a publicly available resource for U.S. population level nutrition data that does not require data use agreements.

### Screener item content validation

An iterative process was used to identify and refine items for the PHD screener. Initial food groups were included based on the components of the HEI-2015 [29, 30], the Alternative Mediterranean Diet [12], Diet Approach to Stop Hypertension (DASH) [17], and the 2020 American Heart Association (AHA) Diet Goals [31] (Table 1).

**Table 1:** Categories of foods in common dietary indexes

Foods	HEI-2015	DASH-Na	AMED	AHA Diet Goals
**Foods to Ingest in Adequate Amounts**				
Fruit	√	√	√	√
Fruit Juice	√	√		
Vegetables ± Potatoes	√	√	√	√
Legumes	√	√	√	√
Plant Proteins	√	√	√	
Nuts/Seeds	√	√	√	
Whole Grains	√	√	√	√
Unsaturated to Saturated Fat Ratio	√		√	
Seafood	√			
Fish	√		√	√
Dairy ± Low-fat	√	√		
**Foods to Ingest Moderately if at All**				
Meat/Poultry/Fish/Eggs		√		
Refined Grains	√	√		
Alcohol			√	
Sodium	√	√		√
Added Sugars	√			
Saturated Fat	√		√	
Sugary Beverages		√		√
Sweets		√		
Fats/Oils		√		
Processed Meat			√	√
Red Meat			√	

*HEI* Healthy Eating Index, *DASH* Diet Approach to Stop Hypertension, *AMED* Alternative Mediterranean Diet, *AHA* American Heart Association

To enhance patient comprehension of the food groups, frequently reported foods and ethnically- or culturally-frequent foods in each screener food group from adult subjects in NHANES 2017–18 [32] were added as examples. As suggested by Bailey [25], foods that subjects typically consider desserts were separated from sweet snacks and breads were separated from cereals to reduce the risk of food omissions. Expert clinical dietitians whose practice included counseling patients with cardiovascular, metabolic, or oncologic disorders added behavioral items often discussed during dietary counseling sessions (adding sugar, salt, or fat). Focus groups (*n* = 7 student participants) and informal feedback from patients (*n* = 10) were used to ensure comprehension of the items.

The screener was made available to four clinical dietitians in paper format and in a REDCap (Vanderbilt University, Nashville, TN) database for electronic data entry by the patient. The time required to complete the PHD screener on paper was observed and recorded by the dietitian, and the time to complete the screener online was determined by subtracting the completion time from the start time in REDCap. The willingness of patients to complete the screener was determined by the response rate to a REDCap invitation by email.

### National Health and Examination Survey

The National Health and Examination Survey (NHANES) is a series of nationally representative studies with the purpose to assess the nutritional intake and health status of adults and children within the U.S. The sample is identified using a multistage, stratified, clustered, probability sampling design with intentional oversampling of Hispanics, non-Hispanic blacks, older adults, and people with low-income. The 2017–2018 NHANES survey in adults age 18+ years was used for this project. The 2015–16 NHANES survey data were used to assess reproducibility of the results of the primary analysis.

The food intake data were obtained from the What We Eat in America (WWEIA) component of the NHANES survey. The WWEIA 24-hour dietary recalls were collected during the visit to the clinical examination center by trained personnel using the validated multi-pass method according to NHANES procedures. The foods and beverages reported in the recalls were distributed into standard servings for 37 food pattern components (FPED) [33] to permit computation of food patterns. Additional food items for the screener were obtained from the Individual Foods File or the Diet Behavior and Nutrition survey.

### Statistical analysis

The individual items in the PHD screener were evaluated by nutrition experts for clarity and relevance to their nutrition counseling practice with the Content Validity Index (CVI) after the approach of Miller [34]. An item-level score was computed based on the clarity (not clear, somewhat clear, quite clear, very clear) or relevance of each (not relevant, somewhat relevant, quite relevant, and highly relevant). The number of experts selecting quite or highly clear (or quite or very relevant) divided by the total number of experts gives the % agreement in a 0–1 range. The mean of all CVI item scores was used to compute the instrument CVI (I-CVI) for the screener. Patient acceptability was assessed by the time required to complete the screener and the completion response rate.

Summary statistics were computed for the demographic characteristics of the NHANES 2017–18 day one dietary recall sample in adults and weighted to account for the complex sampling design of NHANES. Demographic measures were summarized as percentages and means + standard error for categorical and continuous measures, respectively. HEI-2015 scores were computed for the diet recalls. FPED serving counts derived from day one dietary recall data were used to construct simulated individual subject responses to the screener items, using the PHD screener**.** Screener behavioral items with yes/no answers were obtained from the WWEIA Individual Foods File, considering any intake of the food as a yes answer. Intake of fast foods or pizza meals each week was taken from a survey item DBD900 in the Diet Behavior and Nutrition survey with the result divided by 7 to reflect daily intake. Neither diet recall data nor NHANES survey data contains a measure reflective of whether individuals add salt at the table, thus this screener item was excluded from simulation analysis. Simulated screener responses were summarized as frequencies and percentages. The association between each PHD screener item and HEI-2015 total and subcomponent scores from the dietary recall was assessed using Spearman rank-order and rank biserial correlation for continuous and categorical PHD screener items, respectively. Correlations < 0.3 were considered low, 0.3–0.5 moderate, and > 0.5 strong [35]. A sensitivity analysis was conducted to determine whether the associations between simulated screener responses and HEI-2015 variables differed by self-reported racial or ethnic identity of NHANES participants.

The simulated screener items that were strongly or moderately positively associated with HEI variables were assigned a score of 0–5 based on the frequency reported**.** Those with negative associations were reverse scored where 0 servings received 5 points and 5 servings received 0 points. Screener items with a yes/no response were given 1 point for a yes response if the item was positively associated and 0 points if negatively associated with a healthy HEI component score. The screener score was derived by computing the sum of 12 items with values ranging from 0 to 5, and 3 items with values ranging from 0 to 1. The screener total score has a range of 0–63, with higher scores indicating a healthier diet. The HEI-2015 and PHD screener total scores were summarized using median and interquartile range. The association between the PHD screener total score and HEI-2015 total and subcomponents was assessed using Spearman rank-order correlation. The analysis was repeated using NHANES 2015–16 dietary recall data to assess the reproducibility of the results. Statistical analyses were conducted using SAS 9.4 (SAS, Carey, NC). A *p* value< 0.05 was considered statistically significant.

## Results

### Screener development

The PHD screener, consisting of 30 items, is in Table 2.

**Table 2:** Penn healthy diet survey

FOODS YOU DRANK OR ATE YESTERDAY	How Many Times Yesterday?					
**BEVERAGES**	**0**	**1**	**2**	**3**	4	**5 or more**
Water	√	√	√	√	√	√
Coffee or tea	√	√	√	√	√	√
Did you add sugar, honey, or flavored creamers to your coffee or tea?	Yes	No				
Did you add artificial sweeteners to your coffee or tea?	Yes	No				
Did you add half and half or whipped cream to your coffee or tea?	Yes	No				
Sugar-sweetened drinks such as soda, iced tea, sports drinks, fruit drink or fruit punch	√	√	√	√	√	√
Diet soda or artificially-sweetened tea or beverages	√	√	√	√	√	√
Beer, wine, spirits, or wine cooler	√	√	√	√	√	√
Milk	√	√	√	√	√	√
Did you use regular or full fat milk?	Yes	No	Don’t know			
**FRUITS AND VEGETABLES**	**0**	**1**	**2**	**3**	4	**5 or more**
Fruit juice such as orange or apple juice with no added sugar	√	√	√	√	√	√
Fruit (not juice) such as apples, bananas, oranges, tangerines,grapes, or berries	√	√	√	√	√	√
Green or leafy vegetables such as spinach, kale, broccoli, cabbage, cucumber, or salad	√	√	√	√	√	√
Red or orange vegetables such as carrots, tomatoes, peppers, squash, or salsa	√	√	√	√	√	√
**BREAD AND GRAINS**	**0**	**1**	**2**	**3**	4	**5 or more**
Whole grain bread	√	√	√	√	√	√
Cooked whole grains such as oats, quinoa, brown rice, or whole wheat pasta	√	√	√	√	√	√
White bread or rolls, wraps, taco shells, tortillas, burritos, or boxed cereal	√	√	√	√	√	√
Cooked white rice, dumplings, pasta, noodles, grits, baked or boiled potatoes or sweet potatoes but not French fries	√	√	√	√	√	√
Did you add butter or gravy to bread, rolls, biscuits, or potatoes?	Yes	No				
**DAIRY**	**0**	**1**	**2**	**3**	4	**5 or more**
Yogurt	√	√	√	√	√	√
Cheese or queso	√	√	√	√	√	√
**PROTEIN FOODS**	**0**	**1**	**2**	**3**	4	**5 or more**
Eggs	√	√	√	√	√	√
Poultry or chicken that is not fried	√	√	√	√	√	√
Fish or seafood such as shrimp or clams that is not fried	√	√	√	√	√	√
Plant proteins such as beans, peas, lentils, chickpeas, hummus, or tofu	√	√	√	√	√	√
Red meat or pork	√	√	√	√	√	√
Cold cuts, ham, lunchmeats, hot dogs, or kielbasa	√	√	√	√	√	√
Bacon, sausage, or pork roll	√	√	√	√	√	√
Fried foods such as fried chicken, shrimp, fish, eggrolls, rice, or French fries	√	√	√	√	√	√
Fast food meals; Asian takeout; burgers; wings; nachos; or pizza meals	√	√	√	√	√	√
Did you add salt to your food at the table?	yes	no				
Did you add olive oil or vegetable oil (not coconut oil) to foods or use it in cooking?	yes	No	Don’t know			
**SNACKS**	**0**	**1**	**2**	**3**	**4**	**5 or more**
Nuts, seeds, or nut butter	√	√	√	√	√	√
Desserts such as cake, pie, or ice cream	√	√	√	√	√	√
Snacks such as cookies, brownies, donuts, or candy	√	√	√	√	√	√
Salty snacks such as potato, corn, or nacho chips, pretzels, crackers, or popcorn	√	**√**	**√**	**√**	**√**	√

Eleven expert dietitians evaluated the individual screener items for clarity and relevance in response to an anonymous online survey, with free text suggestions to improve the clarity of items. After revision of an item with low clarity (CVI 0.36) and removal of an item with low relevance (CVI 0.73), a subgroup of seven experts responded to the clarity and relevance of the final edited questions. The final PHD individual item clarity CVI ranged 0.81–1.0, and the mean clarity I-CVI for the screener was 0.971 indicating excellent agreement (Table 3). The final PHD individual item CVI for relevance ranged 0.86–1.0, and the mean relevance I-CVI was 0.971, suggesting strong relevance for nutrition counseling. The PHD required a median (IQR) of 4 (3–5) minutes for 10 patients to complete on paper and a median (IQR) of 4 (4–8) minutes for 28 patients to complete online. Seventy-four percent of the first 48 patients who were invited using email completed the survey online. None of 10 patients asked declined to complete the survey on paper.

**Table 3:** Content validity of screener items

Screener Item	Clarity CVI	Relevance CVI
Water (8 oz)	1.0	1.0
Coffee or tea (8 oz)	1.0	0.86
Do you add half and half or whipped cream to your coffee or tea?	1.0	1.0
Do you add sugar, honey, or flavored creamers to your coffee or tea?	1.0	1.0
Do you add artificial sweeteners to your coffee or tea?	1.0	0.86
Sugar-sweetened drinks such as soda, sports drinks, fruit drink or fruit punch (8 oz)	1.0	1.0
Diet soda (8 oz)	1.0	1.0
Protein shakes or supplements (8 oz)	0.81	1.0
Liquid calorie and protein supplements such as Boost or Ensure (8 oz)	0.86	1.0
Beer (12 oz); wine (5 oz), spirits (1-oz shot), or wine cooler (12 oz)	1.0	1.0
Fruit juice with no added sugar (8 oz)	0.91	0.91
Fruit (not juice) such as apples, bananas, oranges, tangerines (piece), or berries (cup)	1.0	1.0
Green, leafy vegetables such as spinach, broccoli, kale, or salad (1 cup cooked or ½ cup raw)	1.0	0.91
Red or orange vegetables such as carrots, tomatoes, peppers, or squash (1 cup cooked or ½ cup raw)	1.0	0.91
Whole grain bread (1 slice)	0.91	1.0
Cooked whole grains such as oats, quinoa, brown rice, or whole wheat pasta (1 cup)	0.91	1.0
White bread (slice); rolls, taco shells, tortillas or burritos (each) or boxed cereal (1 cup)	1.0	1.0
Cooked or fried white rice, dumplings, pasta, noodles, grits, baked or boiled potatoes or sweet potatoes but not French fries (1 cup)	1.0	1.0
Milk (1 cup)	0.91	1.0
Yogurt (1 cup)	0.86	0.86
Cheese (size of a pair of dice) or 1 slice (1 oz)	1.0	1.0
Do you use regular fat milk, yogurt, or cottage cheese?	1.0	0.9
Eggs (each)	1.0	1.0
Poultry such as chicken, turkey, or duck (size of deck of cards or 3 oz) not fried	1.0	1.0
Fish (deck of cards or 3 oz) or shellfish such as shrimp (5–6 pieces or 3 oz) or clams (12 medium or 3 oz) or mussels (25 or 3 oz) not fried	1.0	1.0
Plant proteins such as beans, peas, lentils, chickpeas, hummus, or tofu (1 cup)	1.0	1.0
Red meat or pork (size of deck of cards or 3 oz)	1.0	1.0
Cold cuts or lunchmeat such as ham or bologna (slice), kielbasa or hot dog (each)	1.0	1.0
Nuts or seeds (1 handful or ¼ cup) or nut butter (1 tablespoon)	1.0	1.0
Desserts such as cake or pie, (piece), or ice cream (1/2 cup)	1.0	1.0
Snacks such as cookies, brownies, donuts, or candy bars (each small)	1.0	1.0
Salty snacks such as potato chips (1 oz), nachos/corn chips (10–15), or crackers (6), pretzels (10 mini), or popcorn (3/4 cup)	0.91	0.91
Fried foods such as fried chicken or fish (piece), or French fries (12–15 each)	1.0	1.0
Fast food meals; Asian takeout or fried eggroll; nachos; or pizza (meals each week)	1.0	0.91
Do you use olive oil or vegetable oil (not coconut oil) on foods or in cooking?	1.0	0.91
Do you add butter or gravy to bread, rolls, biscuits, or potatoes?	1.0	1.0
Do you add salt to your food at the table?	1.0	1.0
**Instrument CVI**	**0.971**	**0.971**

*CVI* Content Validity Index, *I-CVI* Instrument Content Validity Index

### Screener items are associated with healthy eating index variables

The demographic and HEI-2015 characteristics of the NHANES 2017–18 sample are in Table 4. The mean age was 48 years, with 52% identified as female, and self-reported racial/ethnic identity as 9% Mexican American, 7% other Hispanic, 62% Non-Hispanic White, 12% Non-Hispanic Black, 6% Non-Hispanic Asian, and 5% other race or multi-racial. The mean body mass index was 29.78 ± 0.28. The mean family income ratio relative to the federal poverty line was 3.04 ± 0.06.

**Table 4:** Demographic characteristics of the National Health and Nutrition Examination Survey 2017–18 adult participants

Variable	Label	Number	Mean	Std Error of Mean	95% CL for Mean	
**Age**	Age in years at screening	4863	47.82	0.63	46.48	49.16
**Income**	Ratio of family income to poverty	4291	3.04	0.06	2.91	3.17
**BMI**	Body Mass Index (kg/m**2)	4806	29.78	0.28	29.19	30.37
**Sex**	**Number**	**Weighted Frequency**		**Row Percent**		
**Male**	2365	116,805,752		48.07		
**Female**	2498	126,187,321		51.93		
**Self-reported racial/ethnic identity**						
**Mexican American**	648	22,046,665		9.07		
**Other Hispanic**	450	17,006,139		6.99		
**Non-Hispanic White**	1735	150,362,288		61.88		
**Non-Hispanic Black**	1150	28,079,784		11.56		
**Non-Hispanic Asian**	635	14,177,595		5.83		
**Other Race - Including Multi-Racial**	245	11,320,602		4.66		
**Total**	4863	242,993,073		100.0000		

**Fig. 1:**
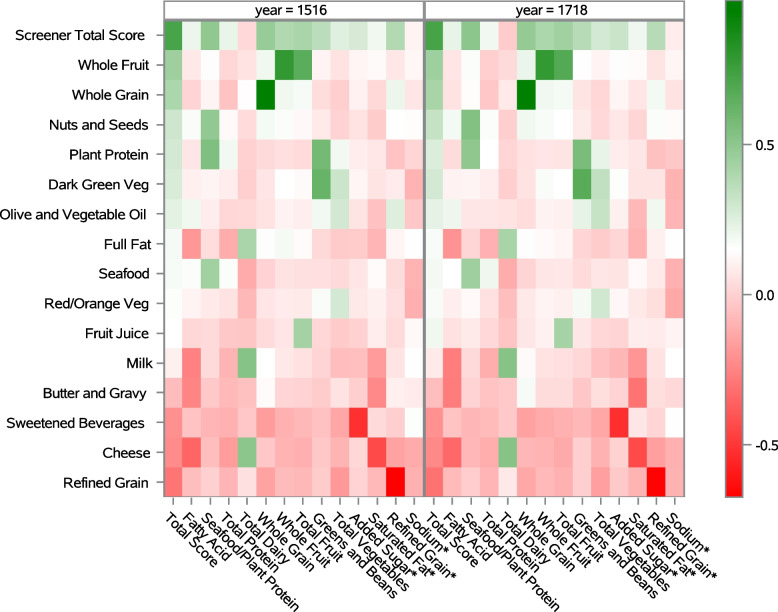
Heat map of Spearman correlations between individual simulated Penn Healthy Diet Screener items (y axis) to Healthy Eating Index (HEI)-2015 components (x axis). Data from adult respondents to the National Health and Nutrition Examination Survey 2015–16 are in the left panel and from the 2017–18 sample are in the right panel. Correlations colored green are positively and red are negatively associated. Abbreviations: HEI = Healthy Eating Index-2015

The NHANES variables used to simulate screener item responses are in Additional file 1, and the PHD items using NHANES food recall data are in Additional file 2. Spearman correlation coefficients of the individual screener items to the total HEI-2015 score and its subcomponents are in Additional file 3 and displayed in the heatmaps in Fig. 1 where the right panel displays NHANES 2017–18 analysis, and the left displays the NHANES 2015–16 reproducibility analysis. The same food groups with strong or moderate positive correlations with HEI-2015 components were identified in both NHANES samples (whole grains, whole fruit, fruit juice, green vegetables, red/orange vegetables, plant proteins, seafood, milk, cheese, nuts/seeds, and oils), and all are scored positively in the HEI-2015. Screener items with strong or moderate negative correlations with HEI components in both samples were refined grains, sugary beverages, cheese, and butter/gravy. The HEI-2015 scores refined grains negatively as items to take in moderation. The sugary beverages item was strongly associated with the negatively scored HEI-2015 added sugar variable, as were cheese and butter/gravy with the negatively scored saturated fat component in HEI-2015. The cheese variable was also negatively associated with the fatty acid ratio (monounsaturated + polyunsaturated fats/saturated fat) in HEI-2015. The HEI-2015 Total score was moderately positively associated with the screener whole fruits, whole grains, nuts/seeds and negatively associated with refined grains. In summary, these findings suggest that the PHD screener can identify pertinent food groups associated with U.S. dietary goals based on the HEI-2015.

### Simple screener scoring algorithm

The screener items that were strongly or moderately positively associated with HEI-2015 components were assigned a score based on the number of servings reported in the predicted screener response (Table 5).

**Table 5:** Penn healthy diet scoring algorithm (score range is 0–63 points, with higher score indicating a healthier diet)

FOODS YOU DRANK OR ATE YESTERDAY	Circle the times you ate this food yesterday					
**Score with 1 point for each serving of food items**	0	1	2	3	4	5 or more
Fruit juice such as orange or apple juice with no added sugar	0	1	2	3	4	5
Fruit such as apples, bananas, oranges, tangerines, or berries (not juice)	0	1	2	3	4	5
Green or leafy vegetables such as spinach, kale, broccoli, cabbage, or salad	0	1	2	3	4	5
Red or orange vegetables such as carrots, tomatoes, peppers, or squash	0	1	2	3	4	5
Whole grain bread + cooked whole grains	0	1	2	3	4	5
Milk	0	1	2	3	4	5
Fish or seafood such as shrimp or clams that is not fried	0	1	2	3	4	5
Plant proteins such as beans, peas, lentils, chickpeas, hummus, or tofu	0	1	2	3	4	5
Nuts, seeds, or nut butter	0	1	2	3	4	5
**Reverse score 1 point for each serving**	0	1	2	3	4	5 or more
Sugar-sweetened drinks such as soda, iced tea, sports drinks, fruit drink or fruit punch	5	4	3	2	1	0
White bread or rolls, wraps, taco shells, tortillas, burritos, or boxed cereal or refined grains	5	4	3	2	1	0
Cheese	5	4	3	2	1	0
**Food behavior questions**						
Did you use regular or full fat milk or yogurt?	Yes = 1	No = 0				
Did you add butter or gravy to bread, rolls, biscuits, or potatoes?	Yes = 0	No = 1				
Did you use olive oil or vegetable oil (not coconut oil) on foods or in cooking?	Yes = 1	No = 0				

**Fig. 2:**
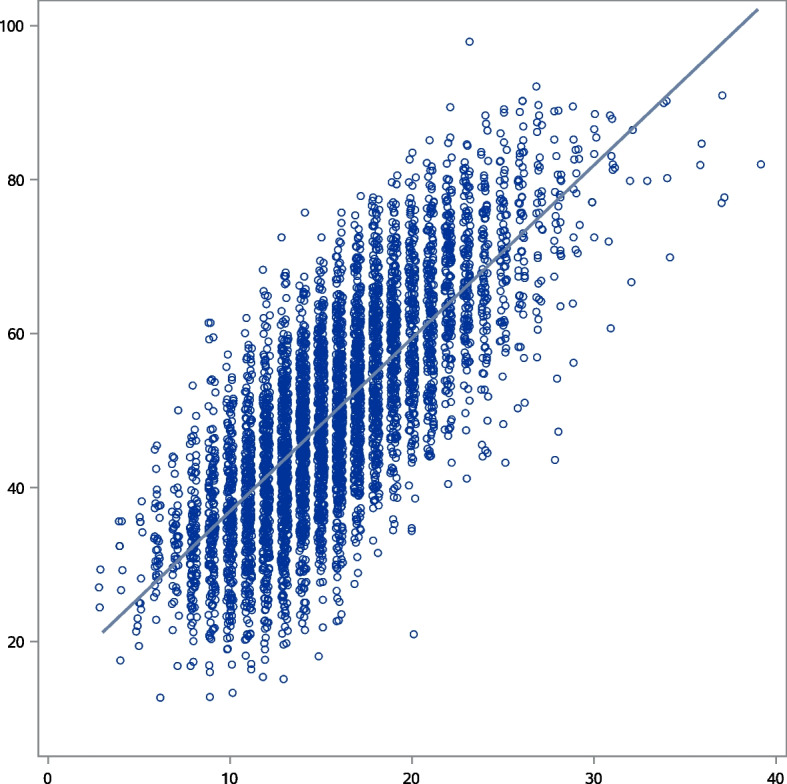
Scatter plot and regression line comparing the simulated Penn Healthy Diet screener score (x-axis) and the Healthy Eating Index (HEI)-2015 score (y-axis) based on 2017–18 National Health and Nutrition Examination Survey (NHANES) in adult participants. The Spearman rho is 0.75

**Fig. 3:**
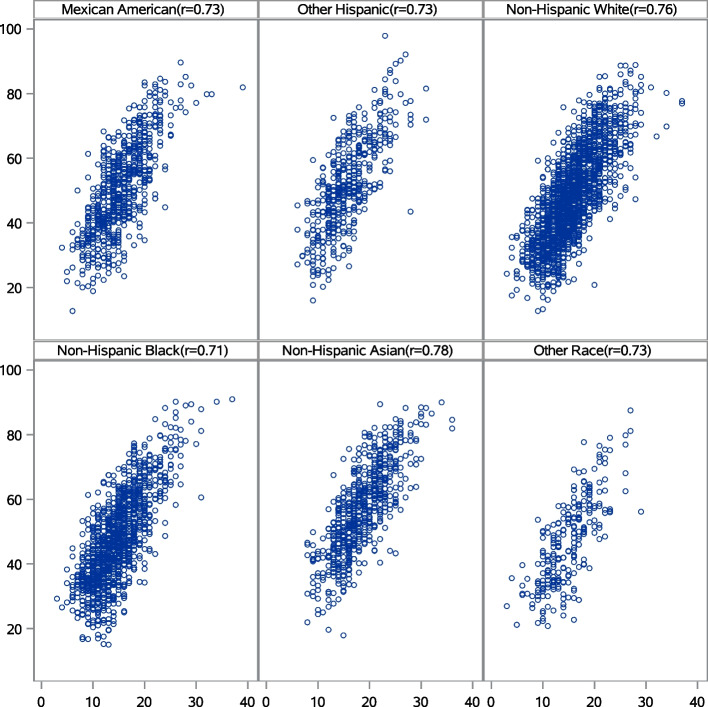
Scatter plots comparing the simulated Penn Healthy Diet screener score on the x-axis and the total Healthy Eating Index (HEI)-2015 score on the y-axis based on 2017–18 National Health and Nutrition Examination Survey (NHANES) in adult participants stratified by self-reported racial/ethnic group. The Spearman rho correlations are listed in each plot

Among NHANES 2017–18 participants, the median (IQR) HEI-2015 score was 48.96 (39.51–59.48) and the simulated PHD median (IQR) score was 14.96 (11.99–18.36). The PHD score was strongly associated (Spearman rho 0.75) with the HEI-2015 score (Fig. 2). The Spearman rho was 0.75 in the reproducibility analysis using the 2015–16 NHANES day one recalls. The HEI-2015 to simulated screener correlations were all > 0.70 across the self-reported racial/ethnic groups designated in NHANES (Fig. 3), suggesting that the screener scoring algorithm can successfully identify a healthy diet according to national dietary guidelines in many US adults.

## Discussion

The Penn Healthy Diet (PHD) screener provides useful, actionable information about dietary intake for nutrition counseling and potentially for research. The PHD is feasible for patient use with low time burden, even when self-administered online. Based on simulated NHANES adult participant PHD item responses, the screener items are largely congruent with the HEI-2015 component scores computed from the dietary recall data, and the simulated PHD score is strongly correlated with HEI-2015 score. Thus, the PHD screener provides a rapid, feasible tool to estimate dietary quality or to begin a diet counseling conversation.

The HEI-2015 measures overall diet quality relative to the Dietary Guidelines for Americans [36] and has been associated with important clinical outcomes such as cardiovascular disease and cancer risk [1, 9, 10]. However, computation of the HEI-2015 score requires knowledge of both servings of foods and nutrient-level intake to compute nutrient density (per 1000 kcal, as a percentage of total kcal, or fatty acid ratio). Such detailed information is not readily available in busy clinical settings. By contrast, the PHD screener captures similar information to the 24-hour recall with low subject burden without the need for sophisticated computerized software for data collection or analysis. Since dietary advice is based on choices of foods rather than the micronutrients they provide, the micronutrient analysis is not helpful for communication to patients. The simple and intuitive scoring algorithm proposed here can be computed in real time, to enable more focused nutrition counseling sessions or a single overall diet quality score. Furthermore, the screener requires only 4 minutes to complete, far less than a 20-minute recall.

To maximize the usefulness of the PHD for counseling relative to the AHA goals listed in Table 1, a second version named Penn Healthy Diet Screener for Dietary Goal Assessment (PHD-G) was created using the same items as the PHD but permitting the identification of daily versus weekly intake of key items (Table 6). This version of the screener requires further validation due to its weekly intake section that was not comparable to NHANES single day recall data. A project comparing the PHD-G responses to usual dietary intake captured by three research dietitian-administered 24-hour recalls in Black women of childbearing age is underway for this purpose.

**Table 6:** Penn Healthy Diet Survey- Goals (PHD-G)

**FOODS YOU DRANK OR ATE YESTERDAY**	**Circle the TIMES YESTERDAY**					
**BEVERAGES**	**0**	**1**	**2**	**3**	4	**5 or more**
Water	√	√	√	√	√	√
Coffee or tea	√	√	√	√	√	√
Did you add sugar, honey, or flavored creamers to your coffee or tea?	Yes	No				
Did you add artificial sweeteners to your coffee or tea?	Yes	No				
Did you add half and half or whipped cream to your coffee or tea?	Yes	No				
Sugar-sweetened drinks such as soda, iced tea, sports drinks, fruit drink or fruit punch	√	√	√	√	√	√
Diet soda or artificially-sweetened tea or beverages	√	√	√	√	√	√
Milk	√	√	√	√	√	√
Did you use regular or full fat milk or yogurt?	Yes	No	**Don’t know**			
**FRUITS AND VEGETABLES**	**0**	**1**	**2**	**3**	4	**5 or more**
Fruit juice such as orange or apple juice with no added sugar	√	√	√	√	√	√
Fruit such as apples, bananas, oranges, tangerines, or berries (not juice)	√	√	√	√	√	√
Green or leafy vegetables such as spinach, kale, broccoli, cabbage, or salad	√	√	√	√	√	√
Red or orange vegetables such as carrots, tomatoes, peppers, or squash	√	√	√	√	√	√
**BREAD AND GRAINS**	**0**	**1**	**2**	**3**	4	**5 or more**
Whole grain bread	√	√	√	√	√	√
Cooked whole grains such as oats, quinoa, brown rice, or whole wheat pasta	√	√	√	√	√	√
White bread or rolls, wraps, taco shells, tortillas, burritos, or boxed cereal	√	√	√	√	√	√
Cooked white rice, dumplings, pasta, noodles, grits, baked or boiled potatoes or sweet potatoes but not French fries	√	√	√	√	√	√
Did you add butter or gravy to bread, rolls, biscuits, or potatoes?	**Yes**	**No**				
**FOODS YOU ATE OR DRANK LAST WEEK**	**Circle the TIMES LAST WEEK**					
**BEVERAGES**	**0**	**1**	**2**	**3**	4	**5 or more**
Beer, wine, spirits, or wine cooler	√	√	√	√	√	√
**DAIRY**	**0**	**1**	**2**	**3**	4	**5 or more**
Yogurt	√	√	√	√	√	√
Cheese	√	√	√	√	√	√
**PROTEIN FOODS**	**0**	**1**	**2**	**3**	4	**5 or more**
Eggs	√	√	√	√	√	√
Poultry or chicken that is not fried	√	√	√	√	√	√
Fish or seafood such as shrimp or clams that is not fried	√	√	√	√	√	√
Plant proteins such as beans, peas, lentils, chickpeas, hummus, or tofu	√	√	√	√	√	√
Red meat or pork	√	√	√	√	√	√
Cold cuts, lunchmeats, hot dogs, or kielbasa	√	√	√	√	√	√
Bacon, sausage, or pork roll	√	√	√	√	√	√
Fried foods such as fried chicken, shrimp, fish, eggrolls, rice, or French fries	√	√	√	√	√	√
Fast food meals; Asian takeout; burgers; wings; nachos; or pizza meals	√	√	√	√	√	√
Did you add salt to your food at the table?	yes	no				
Did you add olive oil or vegetable oil (not coconut oil) to foods or use it in cooking?	yes	No	**Don’t know**			
**SNACKS**	**0**	**1**	**2**	**3**	4	**5 or more**
Nuts, seeds, or nut butter	√	√	√	√	√	√
Desserts such as cake, pie, or ice cream	√	√	√	√	√	√
Snacks such as cookies, brownies, donuts, or candy	√	√	√	√	√	√
Salty snacks such as potato, corn, or nacho chips, pretzels, crackers, or popcorn	√	√	√	√	√	√

This project has strengths and limitations. The PHD screener is focused on foods typically considered part of an optimal diet according to the HEI-2015, the DASH approach, the Mediterranean diet, and clinical experts, with frequently reported examples from NHANES surveys. The PHD scoring algorithm aligns well with HEI-2015 scores. The comparison of simulated PHD screener versus HEI-2015 components was confirmed in two different but recent groups of NHANES respondents, and correlations between PHD item scores and HEI-2015 scores were not significantly different by self-reported racial/ethnic groups in NHANES, suggesting the representative nature of the items. However, the PHD may not be representative of a healthy diet in individuals consuming a more restricted diet such as vegan or other exclusion diets or with those from groups with very different food cultures. While the use of NHANES food recall data to identify common food examples gives a degree of national representativeness to the food examples in the screener, the simulated screener responses were inferred from the food recall data and not made by the participants themselves. Therefore, our hypothetical estimate of their response to some questions is likely biased and overly optimistic. To address this issue and evaluate utility of the screener for omics research, a concurrent validation of the PHD with the Automated Self-Administered 24-hour recall (ASA24) is underway in a large sample of patients deeply phenotyped for Non-alcoholic Fatty Liver Disease (NAFLD).

## Conclusions

In summary, we have developed a new dietary screening tool for use in clinical and potentially in research settings. The PHD screener queries food group exposures in a typical American diet and can be summarized to provide an overall estimate of diet quality that is correlated with HEI-2015 estimates derived from NHANES 24-hour recalls. Evaluation of the utility of the PHD screener for research may demonstrate its potential to provide healthy diet information into precision nutrition research. While the present study establishes the utility and scalability of the PHD screener, and provides a simulated assessment of the instrument’s validity, additional research is needed to better establish validity in reference to a gold standard (diet recall) and to further refine a scoring algorithm.

## Supplementary Information

**Additional file 1: Table 1.** Variables from the National Health and Nutrition Examination Survey used to map to Screener Items.

**Additional file 2: Table 2.** Simulated Screener Item Responses by National Health and Examination Survey 2017–18 Participants.

**Additional file 3: Table 3.** Simulated Screener Response versus Healthy Eating Index-2015 Component Spearman Correlation Coefficients. Bolded items are moderately or strongly associated, all with *p* value< 0.0001. Note that the HEI-2015 index scores moderation components negatively for greater levels of intake.

## Data Availability

The data supporting the conclusions of this article are included within the article and its additional files.
