# Supplementary material for: Development of the Penn Healthy Diet screener with reference to adult dietary intake data from the National Health and Nutrition Examination Survey
Source: Nutr J. 2022 Nov 17;21:70. doi: 10.1186/s12937-022-00821-w (PMC9670424; doi:10.1186/s12937-022-00821-w)
Supplement: Supplementary file 1 — Additional file 1: Table 1. Variables from the National Health and Nutrition Examination Survey used to map to Screener Items. [file 12937_2022_821_MOESM1_ESM.docx]

**Additional Table 1. Variables from the National Health and Nutrition Examination Survey used to map to Screener Items**

| **Screening Variable Name** | **Raw Measure Name(s)** | **Source** | **Labels** | **Notes** | **Unit** |
| --- | --- | --- | --- | --- | --- |
| Alcohol | a_drinks | FPED | Alcoholic beverages |  | # of drinks |
| Fruit Juice | f_juice | FPED | Fruit juices, citrus and non citrus |  | cup eq. |
| Whole Fruit | f_other | FPED | Intact fruits (whole or cut); excluding citrus, melons, and berries |  | cup eq. |
| Green Veg | v_drkgr | FPED | Dark green vegetables |  | cup eq. |
| Red/Orange Veg | v_redor_other | FPED | Other red and orange vegetables, excluding tomatoes and tomato products |  | cup eq. |
| Whole Grains | g_whole | FPED | Whole grains |  | oz. eq. |
| Refined Grains | g_refined | FPED | Refined or non-whole grains |  | oz. eq. |
| Milk | d_milk | FPED | Fluid milk and calcium fortified soy milk |  | cup eq. |
| Yogurt | d_yogurt | FPED | Yogurt |  | cup eq. |
| Cheese | d_cheese | FPED | Cheese |  | cup eq. |
| Eggs | pf_eggs | FPED | Eggs (chicken, duck, goose, quail) and egg substitutes |  | oz. eq. |
| Poultry | pf_poult/3 | FPED | Chicken, turkey, Cornish hens, and game birds; excludes organ meats and cured meat | divided by 3 | oz. eq. |
| Nuts/Seeds | PF_NUTSDS | FPED | Peanuts, tree nuts, and seeds, excludes coconut |  | oz. eq. |
| Meat | pf_meat/3 | FPED | Beef, veal, pork, lamb, game meat; excludes organ meats and cured meat | divided by 3 | oz. eq. |
| Cured Meat | pf_curedmeat/3 | FPED | Cured/luncheon meat made from beef, pork, or poultry | divided by 3 | oz. eq. |
| Fast Foods/Pizza Meals | DBD900_daily | FPED | # of meals from fast food or pizza place |  | (# of meals in the past 7 days)/7 |
| Sugary Beverages | food code 7202 | WWEIA | Soft drinks | sum | # of food items |
|  | food code_7204 | WWEIA | Fruit drinks |  | # of food items |
|  | food code_7206 | WWEIA | Sport and energy drinks |  | # of food items |
|  | food code_7208 | WWEIA | Nutritional beverages |  | # of food items |
|  | food code_7220 | WWEIA | Smoothies and grain drinks |  | # of food items |
| Savory Snacks | food code_5002 | WWEIA | Potato chips | sum | # of food items |
|  | food code_5004 | WWEIA | Tortilla, corn, other chips |  | # of food items |
|  | food code_5006 | WWEIA | Popcorn |  | # of food items |
|  | food code_5008 | WWEIA | Pretzels/snack mix |  | # of food items |
| Coffee/Tea | food code_7302 | WWEIA | Coffee | sum | # of food items |
|  | food code_7304 | WWEIA | Tea |  | # of food items |
| Seafood | PF_SEAFD_HI/3 | FPED | Seafood (finfish, shellfish and other seafood) high in n-3 fatty acids | sum, divided by 3 | oz. eq. |
|  | PF_SEAFD_LOW/3 | FPED | Seafood (finfish, shellfish and other seafood) low in n-3 fatty acids |  | oz. eq. |
| Plant Proteins | PF_SOY | FPED | Soy products, excluding calcium fortified soy milk and immature soybeans | sum | oz. eq. |
|  | PF_LEGUMES | FPED | Legumes computed as protein foods |  | oz. eq. |
| Desserts | food code_5802 | WWEIA | Ice cream and frozen dairy desserts | sum | # of food items |
|  | food code_5804 | WWEIA | Pudding |  | # of food items |
|  | food code_5806 | WWEIA | Gelatins, ices, sorbets |  | # of food items |
|  | food code_5502 | WWEIA | Cakes and pies |  | # of food items |
|  | food code_5504 | WWEIA | Cookies and brownies |  | # of food items |
|  | food code_5506 | WWEIA | Doughnuts, sweet rolls, pastries |  | # of food items |
| Sugar/Honey | food code_8008 | WWEIA | Cream and cream substitutes | sum | # of food items |
|  | food code_8802 | WWEIA | Sugars and honey |  | # of food items |
| Artificial Sweetener | food code_8804 | WWEIA | Sugar substitutes |  | # of food items |
| Creamer | food code_8008 | WWEIA | Cream and cream substitutes |  | # of food items |
| Diet Soda | food code_7102 | WWEIA | Diet soft drinks |  | # of food items |
| Full Fat | food code_1002 | WWEIA | Milk, whole | sum | # of food items |
|  | food code_1820 | WWEIA | Yogurt, regular |  | # of food items |
|  | food code_1822 | WWEIA | Yogurt, Greek |  | # of food items |
|  | food code_1604 | WWEIA | Cottage/ricotta cheese |  | # of food items |
| Butter/Gravy | food code_8002 | WWEIA | Butter and animal fats |  | # of food items |
| Oils | food code_8012 | WWEIA | Salad dressings and vegetable oils |  | # of food items |
