# Supplementary material for: Development of the Penn Healthy Diet screener with reference to adult dietary intake data from the National Health and Nutrition Examination Survey
Source: Nutr J. 2022 Nov 17;21:70. doi: 10.1186/s12937-022-00821-w (PMC9670424; doi:10.1186/s12937-022-00821-w)
Supplement: Supplementary file 2 — Additional file 2: Table 2. Simulated Screener Item Responses by National Health and Examination Survey 2017–18 Participants. [file 12937_2022_821_MOESM2_ESM.docx]

**Additional Table 2. Simulated Screener Item Responses by National Health and Examination Survey 2017-18 Participants**

| **Variable** | **Response Category** | **Unweighted Frequency** | **Weighted Frequency** | **Standard Error of Wgt Freq** | **Weighted Percent** | **Standard Error of Weighted Percent** |
| --- | --- | --- | --- | --- | --- | --- |
| Coffee or Tea | 0 | 1549 | 69090190 | 3394582 | 28.44 | 0.97 |
| Coffee or Tea | 2 | 1999 | 100873118 | 4208000 | 41.52 | 1.05 |
| Coffee or Tea | 3 | 902 | 49190826 | 2976151 | 20.25 | 0.94 |
| Coffee or Tea | 4 | 304 | 18327944 | 1584916 | 7.54 | 0.57 |
| Coffee or Tea | 5 | 108 | 5441731 | 663280.5 | 2.24 | 0.27 |
| Alcoholic Drink | 0 | 3880 | 186631107 | 7321077 | 76.83 | 1.35 |
| Alcoholic Drink | 1 | 148 | 6211291 | 752158.3 | 2.56 | 0.33 |
| Alcoholic Drink | 2 | 291 | 19356832 | 2170336 | 7.97 | 0.82 |
| Alcoholic Drink | 3 | 181 | 9492839 | 1444394 | 3.91 | 0.55 |
| Alcoholic Drink | 4 | 106 | 7212753 | 1033575 | 2.97 | 0.42 |
| Alcoholic Drink | 5 | 256 | 14018986 | 1083762 | 5.77 | 0.42 |
| Fruit Juice | 0 | 3314 | 169187957 | 7838263 | 69.65 | 1.35 |
| Fruit Juice | 1 | 1162 | 56746718 | 3274627 | 23.36 | 1.54 |
| Fruit Juice | 2 | 269 | 12213659 | 1439539 | 5.03 | 0.49 |
| Fruit Juice | 3 | 71 | 3062344 | 475883.8 | 1.26 | 0.19 |
| Fruit Juice | 4 | 25 | 950994 | 269366.4 | 0.39 | 0.11 |
| Fruit Juice | 5 | 21 | 762136 | 294286.2 | 0.31 | 0.12 |
| Sugary Beverages | 0 | 2632 | 141463937 | 6303444 | 58.23 | 1.59 |
| Sugary Beverages | 2 | 1436 | 67303935 | 3217870 | 27.71 | 1.12 |
| Sugary Beverages | 3 | 555 | 23383309 | 2352678 | 9.63 | 0.86 |
| Sugary Beverages | 4 | 180 | 7125201 | 1212126 | 2.93 | 0.47 |
| Sugary Beverages | 5 | 59 | 3647427 | 655408.3 | 1.50 | 0.25 |
| Diet Soda | 0 | 4541 | 223341750 | 7649684 | 91.94 | 0.57 |
| Diet Soda | 1 | 230 | 13774542 | 1397127 | 5.67 | 0.44 |
| Diet Soda | 2 | 57 | 3072161 | 757815.6 | 1.26 | 0.31 |
| Diet Soda | 3 | 24 | 2100947 | 425346.8 | 0.86 | 0.17 |
| Diet Soda | 4 | 7 | 567101 | 270198.3 | 0.23 | 0.11 |
| Diet Soda | 5 | 3 | 67308 | 47227.93 | 0.03 | 0.02 |
| Eggs | 0 | 1404 | 68936829 | 3237640 | 28.38 | 0.83 |
| Eggs | 1 | 2197 | 114449979 | 4587014 | 47.11 | 0.92 |
| Eggs | 2 | 547 | 24314457 | 1486639 | 10.01 | 0.66 |
| Eggs | 3 | 487 | 24060324 | 1937776 | 9.90 | 0.63 |
| Eggs | 4 | 152 | 7174027 | 970035.8 | 2.95 | 0.38 |
| Eggs | 5 | 75 | 3988192 | 844721.8 | 1.64 | 0.33 |
| Poultry | 0 | 2706 | 135908480 | 5110548 | 55.95 | 1.52 |
| Poultry | 1 | 1095 | 56444605 | 3608128 | 23.24 | 0.94 |
| Poultry | 2 | 698 | 33223412 | 2722288 | 13.68 | 0.92 |
| Poultry | 3 | 237 | 12064802 | 1375748 | 4.97 | 0.56 |
| Poultry | 4 | 75 | 3256028 | 587076.8 | 1.34 | 0.24 |
| Poultry | 5 | 51 | 2026481 | 350084.0 | 0.83 | 0.15 |
| Nuts and Seeds | 0 | 3131 | 144391597 | 7156247 | 59.44 | 1.74 |
| Nuts and Seeds | 1 | 825 | 48525836 | 1839066 | 19.98 | 0.61 |
| Nuts and Seeds | 2 | 293 | 15430430 | 2127582 | 6.35 | 0.86 |
| Nuts and Seeds | 3 | 240 | 13722526 | 1453327 | 5.65 | 0.56 |
| Nuts and Seeds | 4 | 124 | 6418128 | 1131557 | 2.64 | 0.45 |
| Nuts and Seeds | 5 | 249 | 14435291 | 1738495 | 5.94 | 0.71 |
| Beef and Pork | 0 | 2541 | 123812591 | 5324661 | 50.97 | 2.35 |
| Beef and Pork | 1 | 1423 | 72040231 | 5385585 | 29.66 | 1.52 |
| Beef and Pork | 2 | 613 | 32162761 | 2819214 | 13.24 | 0.93 |
| Beef and Pork | 3 | 178 | 9743369 | 1279161 | 4.01 | 0.49 |
| Beef and Pork | 4 | 55 | 2686905 | 416619.6 | 1.11 | 0.17 |
| Beef and Pork | 5 | 52 | 2477953 | 634235.7 | 1.02 | 0.25 |
| Cured Meat | 0 | 2736 | 131653337 | 4809468 | 54.20 | 1.04 |
| Cured Meat | 1 | 1654 | 83589373 | 4621606 | 34.41 | 1.01 |
| Cured Meat | 2 | 372 | 22063862 | 1294346 | 9.08 | 0.49 |
| Cured Meat | 3 | 79 | 4778811 | 933182.0 | 1.97 | 0.40 |
| Cured Meat | 4 | 13 | 506064 | 190559.3 | 0.21 | 0.08 |
| Cured Meat | 5 | 8 | 332362 | 122379.0 | 0.14 | 0.05 |
| Pizza or Fast Food Meals per week | 0 | 1032 | 56322114 | 3581615 | 27.62 | 1.74 |
| Pizza or Fast Food Meals per week | 1 | 2544 | 134034064 | 6517248 | 65.74 | 1.31 |
| Pizza or Fast Food Meals per week | 2 | 176 | 10644668 | 1654800 | 5.22 | 0.74 |
| Pizza or Fast Food Meals per week | 3 | 55 | 2575315 | 486498.2 | 1.26 | 0.22 |
| Pizza or Fast Food Meals per week | 4 | 8 | 319916 | 154056.2 | 0.16 | 0.08 |
| Savory Snacks | 0 | 3369 | 161889358 | 6154272 | 66.64 | 1.32 |
| Savory Snacks | 2 | 1268 | 68875645 | 3938130 | 28.35 | 1.24 |
| Savory Snacks | 3 | 187 | 9384461 | 1419734 | 3.86 | 0.54 |
| Savory Snacks | 4 | 34 | 2569020 | 751616.8 | 1.06 | 0.31 |
| Savory Snacks | 5 | 4 | 205326 | 115753.6 | 0.08 | 0.05 |
| Fish or Shellfish | 0 | 3976 | 202855642 | 8552515 | 83.51 | 0.96 |
| Fish or Shellfish | 1 | 505 | 21327360 | 1897001 | 8.78 | 0.77 |
| Fish or Shellfish | 2 | 217 | 11606847 | 952482.3 | 4.78 | 0.43 |
| Fish or Shellfish | 3 | 83 | 3815225 | 397926.1 | 1.57 | 0.19 |
| Fish or Shellfish | 4 | 37 | 1671955 | 748216.5 | 0.69 | 0.31 |
| Fish or Shellfish | 5 | 44 | 1646780 | 298687.2 | 0.68 | 0.12 |
| Whole Fruit | 0 | 2629 | 133721811 | 7232927 | 55.05 | 1.96 |
| Whole Fruit | 1 | 1444 | 73190967 | 4423571 | 30.13 | 1.72 |
| Whole Fruit | 2 | 515 | 23559415 | 2358679 | 9.70 | 0.89 |
| Whole Fruit | 3 | 174 | 8095098 | 1107751 | 3.33 | 0.45 |
| Whole Fruit | 4 | 67 | 2856871 | 401360.2 | 1.18 | 0.16 |
| Whole Fruit | 5 | 33 | 1499647 | 344573.3 | 0.62 | 0.13 |
| Plant Proteins | 0 | 3502 | 172630243 | 5111501 | 71.06 | 1.41 |
| Plant Proteins | 1 | 497 | 29064735 | 2925741 | 11.96 | 0.87 |
| Plant Proteins | 2 | 296 | 14955885 | 991404.5 | 6.16 | 0.47 |
| Plant Proteins | 3 | 214 | 9996459 | 1169961 | 4.12 | 0.47 |
| Plant Proteins | 4 | 133 | 6941502 | 1680399 | 2.86 | 0.63 |
| Plant Proteins | 5 | 220 | 9334984 | 1602391 | 3.84 | 0.63 |
| Desserts | 0 | 2632 | 132199926 | 4937491 | 54.42 | 1.18 |
| Desserts | 2 | 1586 | 77642187 | 4254977 | 31.96 | 1.05 |
| Desserts | 3 | 491 | 26149709 | 1428605 | 10.76 | 0.51 |
| Desserts | 4 | 106 | 4337713 | 502111.4 | 1.79 | 0.20 |
| Desserts | 5 | 47 | 2594273 | 579132.9 | 1.07 | 0.23 |
| Dark Green Vegetables | 0 | 3668 | 179978804 | 7409555 | 74.09 | 1.25 |
| Dark Green Vegetables | 1 | 958 | 50417347 | 2838289 | 20.75 | 1.08 |
| Dark Green Vegetables | 2 | 191 | 9614957 | 970522.6 | 3.96 | 0.38 |
| Dark Green Vegetables | 3 | 34 | 2051207 | 678614.8 | 0.84 | 0.28 |
| Dark Green Vegetables | 4 | 6 | 243383 | 118970.6 | 0.10 | 0.05 |
| Dark Green Vegetables | 5 | 5 | 618109 | 486002.6 | 0.25 | 0.20 |
| Red or Orange Vegetables | 0 | 3060 | 152404442 | 6794305 | 62.74 | 1.91 |
| Red or Orange Vegetables | 1 | 1715 | 85345765 | 5482254 | 35.13 | 1.77 |
| Red or Orange Vegetables | 2 | 75 | 4894842 | 925036.4 | 2.01 | 0.37 |
| Red or Orange Vegetables | 3 | 9 | 207389 | 71400.31 | 0.09 | 0.03 |
| Red or Orange Vegetables | 4 | 3 | 71370 | 41500.61 | 0.03 | 0.02 |
| Whole Grains | 0 | 2581 | 126966834 | 5174188 | 52.27 | 1.37 |
| Whole Grains | 1 | 904 | 48203735 | 2593862 | 19.84 | 0.79 |
| Whole Grains | 2 | 656 | 31829625 | 2559313 | 13.10 | 0.81 |
| Whole Grains | 3 | 372 | 18021537 | 1355830 | 7.42 | 0.56 |
| Whole Grains | 4 | 169 | 8800073 | 1090008 | 3.62 | 0.40 |
| Whole Grains | 5 | 180 | 9102003 | 1174389 | 3.75 | 0.47 |
| Refined Grains | 0 | 131 | 6919958 | 1227797 | 2.85 | 0.49 |
| Refined Grains | 1 | 222 | 10677433 | 1082855 | 4.40 | 0.41 |
| Refined Grains | 2 | 446 | 23459777 | 1681276 | 9.66 | 0.54 |
| Refined Grains | 3 | 513 | 25906524 | 2033696 | 10.66 | 0.66 |
| Refined Grains | 4 | 583 | 26538844 | 1902832 | 10.92 | 0.66 |
| Refined Grains | 5 | 2967 | 149421272 | 5242269 | 61.51 | 1.16 |
| Milk | 0 | 1207 | 55622832 | 3966177 | 22.90 | 1.30 |
| Milk | 1 | 2593 | 134549825 | 5078530 | 55.39 | 1.09 |
| Milk | 2 | 698 | 34573519 | 2297895 | 14.23 | 0.75 |
| Milk | 3 | 237 | 11644543 | 1028742 | 4.79 | 0.41 |
| Milk | 4 | 68 | 3365620 | 634792.0 | 1.39 | 0.27 |
| Milk | 5 | 59 | 3167469 | 583603.8 | 1.30 | 0.23 |
| Yogurt | 0 | 4396 | 216327516 | 7904313 | 89.05 | 1.00 |
| Yogurt | 1 | 428 | 23819083 | 2501353 | 9.81 | 0.94 |
| Yogurt | 2 | 34 | 2485597 | 577537.2 | 1.02 | 0.24 |
| Yogurt | 3 | 3 | 280748 | 145339.0 | 0.12 | 0.06 |
| Yogurt | 5 | 1 | 10865 | 10865.16 | 0.00 | 0.00 |
| Cheese | 0 | 1856 | 76801295 | 2587085 | 31.62 | 0.92 |
| Cheese | 1 | 1864 | 99259227 | 4988161 | 40.86 | 0.90 |
| Cheese | 2 | 766 | 43904346 | 2292727 | 18.07 | 0.92 |
| Cheese | 3 | 239 | 14644377 | 1938211 | 6.03 | 0.72 |
| Cheese | 4 | 82 | 4574152 | 587535.2 | 1.88 | 0.24 |
| Cheese | 5 | 55 | 3740411 | 1078290 | 1.54 | 0.42 |
| Added Sugar or Honey | No | 2848 | 143430654 | 6780176 | 59.04 | 1.70 |
| Added Sugar or Honey | Yes | 2014 | 99493154 | 5253666 | 40.96 | 1.70 |
| Artificial Sweetener | No | 4486 | 225561740 | 7710406 | 92.85 | 0.69 |
| Artificial Sweetener | Yes | 376 | 17362069 | 1940771 | 7.15 | 0.69 |
| Cream or half and half | No | 3654 | 180749607 | 6122485 | 74.41 | 1.16 |
| Cream or half and half | Yes | 1208 | 62174202 | 4105451 | 25.59 | 1.16 |
| Full Fat Dairy | No | 4028 | 198543719 | 7610462 | 81.73 | 1.10 |
| Full Fat Dairy | Yes | 834 | 44380089 | 3068940 | 18.27 | 1.10 |
| Added Butter or Gravy | No | 4346 | 211930452 | 6754779 | 87.24 | 0.92 |
| Added Butter or Gravy | Yes | 516 | 30993357 | 2918856 | 12.76 | 0.92 |
| Olive or Vegetable Oil | No | 4062 | 193483186 | 6463586 | 79.65 | 1.14 |
| Olive or Vegetable Oil | Yes | 800 | 49440622 | 3759787 | 20.35 | 1.14 |
|  |  |  |  |  |  |  |
