# Supplementary material for: Development of the Penn Healthy Diet screener with reference to adult dietary intake data from the National Health and Nutrition Examination Survey
Source: Nutr J. 2022 Nov 17;21:70. doi: 10.1186/s12937-022-00821-w (PMC9670424; doi:10.1186/s12937-022-00821-w)
Supplement: Supplementary file 3 — Additional file 3: Table 3. Simulated Screener Response versus Healthy Eating Index-2015 Component Spearman Correlation Coefficients. Bolded items are moderately or strongly associated, all with p value< 0.0001. Note that the HEI-2015 index scores moderation components negatively for greater levels of intake. [file 12937_2022_821_MOESM3_ESM.docx]

**Additional Table 3. Simulated Screener Response versus Healthy Eating Index-2015 Component Spearman Correlation Coefficients. Bolded items are moderately or strongly associated, all with p value<0.0001. Note that the HEI-2015 index scores moderation components negatively for greater levels of intake.**

|  |  |  | **Healthy Eating Index-2015**  **Adequacy Components** | | | |  |  |  | **Healthy Eating Index-2015 Moderation Components** | | | | **HEI-2015 Score** |
| --- | --- | --- | --- | --- | --- | --- | --- | --- | --- | --- | --- | --- | --- | --- |
| **Screener Item** | **Total Fruit** | **Whole Fruit** | **Total Vegetables** | **Greens & Beans** | **Whole Grains** | **Total Dairy** | **Total Protein** | **Seafood**  **& Plant Proteins** | **Fatty Acid Ratio** | **Sodium** | **Refined Grains** | **Saturated Fats** | **Added Sugars** | **HEI-2015 Total** |
| Coffee or Tea | 0.07 | 0.11 | 0.09 | 0.08 | 0.13 | 0.02 | 0.03 | 0.10 | 0.01 | 0.05 | 0.00 | -0.03 | 0.02 | 0.12 |
| Sugary Beverages | -0.10 | -0.12 | -0.13 | -0.08 | -0.15 | -0.02 | -0.07 | -0.08 | -0.04 | 0.15 | 0.02 | 0.07 | **-0.52** | -0.20 |
| Diet Soda | -0.02 | -0.01 | -0.01 | -0.04 | 0.02 | 0.03 | 0.00 | -0.02 | -0.02 | -0.07 | -0.04 | -0.09 | 0.10 | -0.03 |
| Alcohol | -0.08 | -0.09 | -0.06 | 0.01 | -0.07 | -0.07 | -0.01 | -0.01 | 0.00 | 0.13 | 0.14 | 0.10 | 0.12 | 0.05 |
| Fruit Juice | **0.44** | 0.11 | 0.02 | 0.07 | 0.07 | -0.04 | 0.03 | 0.09 | 0.06 | 0.12 | 0.09 | 0.10 | 0.01 | 0.20 |
| Whole Fruit | **0.71** | **0.82** | 0.12 | 0.16 | 0.22 | 0.03 | -0.01 | 0.17 | 0.07 | 0.12 | 0.07 | 0.14 | 0.16 | **0.47** |
| Green Vegetables | 0.16 | 0.17 | **0.35** | **0.70** | 0.06 | 0.00 | 0.09 | 0.12 | 0.11 | -0.10 | 0.07 | 0.07 | 0.16 | 0.29 |
| Red/orange Vegetables | 0.10 | 0.11 | **0.31** | 0.19 | 0.08 | -0.06 | 0.05 | 0.13 | 0.10 | -0.13 | 0.05 | 0.08 | 0.13 | 0.18 |
| Whole Grains | 0.19 | 0.20 | 0.02 | 0.07 | **0.98** | 0.08 | -0.03 | 0.14 | 0.06 | 0.06 | 0.19 | 0.07 | 0.12 | **0.44** |
| Refined Grains | -0.10 | -0.07 | -0.16 | 0.00 | -0.13 | 0.08 | -0.09 | -0.01 | -0.07 | -0.10 | **-0.67** | -0.09 | -0.02 | **-0.30** |
| Milk | 0.05 | 0.06 | -0.05 | 0.02 | 0.14 | **0.54** | -0.11 | 0.03 | -0.27 | 0.15 | 0.06 | -0.19 | -0.08 | 0.09 |
| Yogurt | 0.18 | 0.21 | 0.08 | 0.09 | 0.16 | 0.17 | -0.04 | 0.09 | -0.01 | 0.06 | 0.04 | 0.06 | 0.09 | 0.22 |
| Cheese | -0.12 | -0.09 | -0.10 | 0.00 | -0.08 | **0.54** | -0.11 | -0.08 | **-0.34** | -0.10 | -0.16 | **-0.43** | 0.01 | -0.23 |
| Eggs | 0.02 | 0.02 | -0.02 | 0.04 | 0.00 | -0.04 | 0.20 | 0.02 | 0.09 | -0.04 | 0.05 | -0.15 | 0.08 | 0.03 |
| Poultry | -0.01 | -0.03 | -0.01 | 0.01 | -0.05 | -0.11 | 0.24 | -0.10 | 0.17 | -0.13 | 0.01 | 0.07 | 0.04 | 0.01 |
| Seafood | 0.08 | 0.07 | 0.07 | 0.04 | 0.01 | -0.12 | 0.21 | **0.47** | 0.15 | -0.10 | 0.08 | 0.13 | 0.07 | 0.19 |
| Plant Proteins | 0.07 | 0.07 | 0.23 | **0.58** | 0.05 | 0.02 | 0.15 | **0.52** | 0.04 | -0.03 | -0.05 | 0.07 | 0.09 | 0.27 |
| Meat | -0.09 | -0.08 | 0.04 | 0.03 | -0.14 | -0.01 | 0.17 | -0.08 | -0.17 | -0.02 | 0.02 | -0.15 | 0.00 | -0.14 |
| Cured Meat | -0.10 | -0.10 | -0.15 | -0.09 | -0.03 | 0.06 | 0.11 | -0.12 | -0.07 | -0.23 | -0.01 | -0.22 | -0.02 | -0.21 |
| Fast Food or Pizza Meals | -0.17 | -0.17 | -0.14 | -0.11 | -0.12 | 0.00 | -0.04 | -0.12 | -0.09 | -0.01 | -0.07 | -0.08 | -0.16 | -0.23 |
| Nuts & Seeds | 0.15 | 0.18 | 0.03 | 0.09 | 0.20 | 0.00 | 0.17 | **0.56** | 0.19 | 0.14 | 0.17 | 0.02 | 0.07 | **0.34** |
| Desserts | 0.03 | 0.06 | -0.05 | 0.01 | 0.01 | 0.05 | -0.11 | 0.01 | -0.12 | 0.16 | -0.06 | -0.18 | -0.21 | -0.09 |
| Savory Snacks | -0.03 | -0.02 | 0.00 | -0.03 | 0.12 | -0.01 | -0.08 | -0.04 | 0.12 | 0.01 | 0.00 | -0.01 | -0.01 | 0.02 |
|  |  |  |  |  |  |  |  |  |  |  |  |  |  |  |
| Artificial Sweeteners | 0.09 | 0.11 | 0.07 | 0.04 | 0.17 | -0.02 | 0.11 | 0.13 | 0.05 | -0.05 | 0.12 | -0.07 | 0.20 | 0.18 |
| Butter or Gravy | 0.04 | 0.04 | 0.04 | -0.03 | 0.18 | -0.02 | -0.04 | 0.01 | -0.27 | 0.03 | 0.05 | **-0.30** | 0.01 | -0.06 |
| Half and Half or Creamer | -0.01 | 0.01 | -0.03 | 0.00 | 0.03 | -0.07 | 0.03 | 0.05 | 0.01 | 0.05 | 0.03 | -0.09 | -0.03 | 0.00 |
| Full Fat Dairy | 0.12 | 0.13 | -0.01 | 0.02 | 0.16 | **0.44** | -0.10 | 0.02 | -0.20 | 0.15 | 0.10 | -0.09 | 0.02 | 0.18 |
| Oils | 0.10 | 0.11 | **0.34** | 0.23 | 0.04 | 0.06 | 0.07 | 0.07 | 0.21 | -0.09 | 0.20 | -0.08 | 0.10 | 0.24 |
| Added Sugar or Honey | 0.04 | 0.05 | 0.01 | 0.05 | 0.08 | -0.02 | 0.00 | 0.08 | 0.01 | 0.09 | -0.03 | 0.00 | -0.05 | 0.06 |
| Screener Score | **0.46** | **0.42** | 0.29 | **0.39** | **0.49** | -0.02 | 0.20 | **0.53** | 0.22 | 0.09 | **0.39** | 0.20 | **0.32** | **0.75** |
